# Abdominoperineal Approach to Uterovaginal Anastomosis in Cervical Dysgenesis: A Case Report and Review of Literature

**DOI:** 10.1055/s-0042-1757555

**Published:** 2022-11-06

**Authors:** Ajay Halder, Shweta Patel, Pankhuri Dubey

**Affiliations:** 1 Department of Obstetrics and Gynecology, All India Institute of Medical Sciences, Bhopal, Madhya Pradesh, India

**Keywords:** uterovaginal anastomosis, cervical dysgenesis, Mullerian anomalies, primary amenorrhea

## Abstract

Genital outflow tract obstruction due to cervical agenesis is an uncommon Mullerian duct anomaly, increasingly being treated with conservative surgery by creation of an outflow tract by drilling or coring into the cervical remnant or by uterovaginal anastomosis. A 19-year-old woman with cervical dysgenesis in the present case underwent a successful uterovaginal anastomosis to relieve the obstructive menstrual symptoms and preserve the future reproductive function. The neouterovaginal canal was created over a mold of Foley's catheter by anastomosis anterior surface of the uterine corpus to the vaginal vault, bypassing the dysgenetic cervix and using the fibrous band of cervix as support. Normal cyclical menses were restored. Steps of the procedure are detailed in this case report.

Mullerian duct anomalies affect 1 to 4% of the general population, of which congenital cervical anomaly represents a rare variant. 1 These anomalies cause an obstruction of the genital outflow tract either due to the complete absence of cervix or its defective development, with known morphological variants ranging from rudimentary fibrous cord to multiple fragmented segments. 2 Clinically, adolescent girls present with amenorrhea, cyclical pelvic pain with varying degrees of pelvic organ enlargement due to hematometra and/or hematosalpinx, with or without other adnexal pathology following ensuing endometriosis. Early diagnosis and treatment are essential to prevent extensive endometriosis, which may lead to irreversible damage to reproductive potential and necessitate aggressive surgery such as hysterectomy and/or adnexectomy. 3

We present a case with cervical dysgenesis in a 19-year-old woman who was treated with surgical construction of a genital outflow tract by a novel uterovaginal anastomosis technique using the fibrous cervical band as a scaffold for neocervix.

## Case Report

**Fig. 1:**
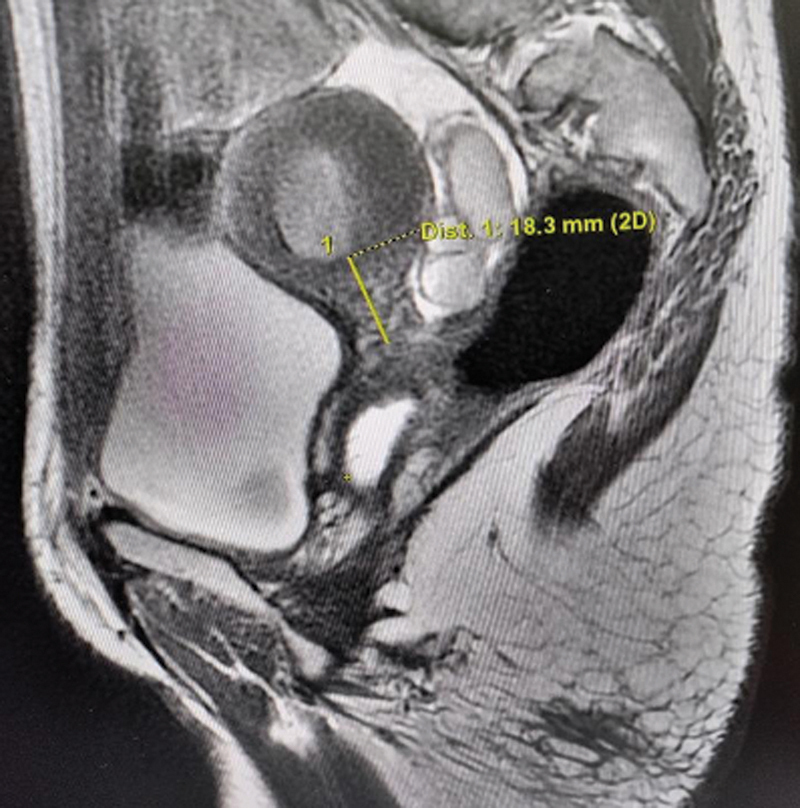
T2-weighted magnetic resonance imaging showing hematometra with an 18.3-mm long noncanalized cervix. Saline was injected in the vagina prior to imaging to delineate the cervicovaginal relationship.

A 19-year-old sexually active woman with primary amenorrhea presented with cyclical pelvic pain for 3 years. On examination, secondary sexual characters were well developed; breasts and pubic hair were Tanner Stages 3 and 4, respectively. No mass was palpable on abdominal examination. External genitalia was normal female type with a proximally blind but otherwise patent vagina of 8 cm. A normal-sized uterus could be palpated on bimanual examination through the blind vagina; however, the cervix was not discernible. Outflow tract obstruction at the level of the cervix was therefore suspected clinically. Imaging showed a normal-sized uterus (7.7 × 5.1 × 3.3 cm) with a hypointense endometrial collection (1.8 × 2.6 × 2.6 cm) suggestive of hematometra and left-sided hematosalpinx. An 18-mm long cervix was identifiable on magnetic resonance imaging (MRI), but the endocervical canal was not visualized ( Fig. 1 ). Vagina, ovaries, bilateral kidneys, and ureters were normal. Patient expressed a desire to restore her menstrual function.

**Fig. 2:**
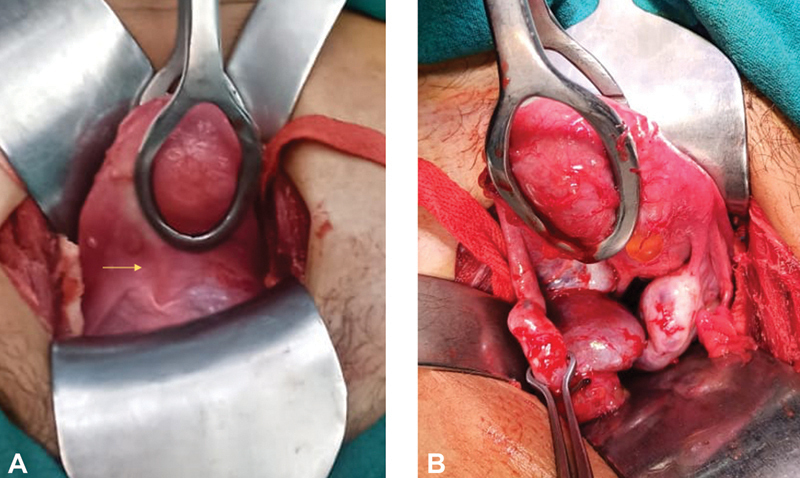
Intraoperative images. ( **A** ) Intraoperative image of the anomalous cervix in the form of a fibrous raphe (marked by the yellow arrow), attached to a normal uterus. ( **B** ) Intraoperative image of uterus and bilateral fallopian tubes and ovaries (posterior view). Left ovary is bulky with hematosalpinx on the same side. Right ovary and tube are normal.

Surgery was planned via abdominoperineal approach. Intraoperatively, the uterus was small and the cervix was palpable as a small fibrous raphe, confirming outflow obstruction at the level of the cervix. Left fallopian tube was dilated and tortuous with hemorrhagic content within. The right tube did not show evidence of any retrograde collection. Both ovaries were normal ( Fig. 2 ). It was evident at the foresight that drilling or coring to create a neocervical canal would not be possible as the cervical remnant was small and nodule-like, with a maximum diameter of 3 mm. Uterovaginal anastomosis was therefore performed, bypassing the atretic cervix, in an attempt to relieve the retrograde menstrual symptoms and restore the genital tract patency. In a novel approach, the fibrous raphe was used as a scaffold for the neouterovaginal anastomosis.

**Fig. 3:**
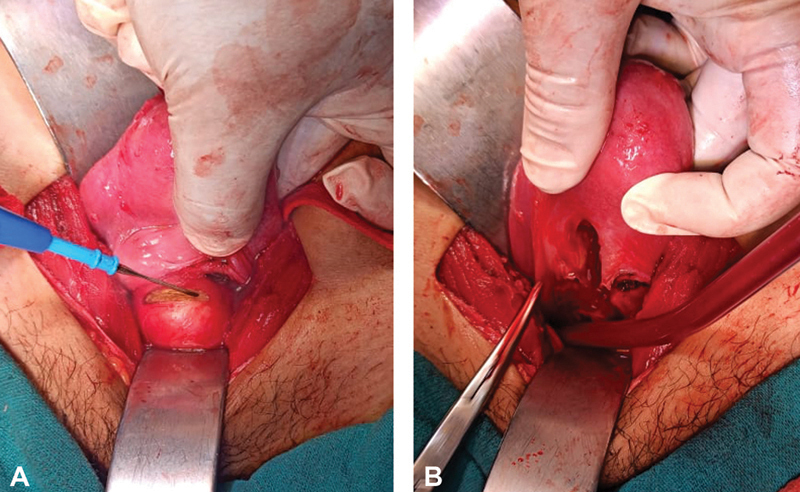
Creation of stomas on ( **A** ) the vaginal vault and ( **B** ) on the uterine corpus.

The vesicouterine space was dissected to expose the lower end of the uterus, the rudimentary cervix, and the vault of the blind vagina. Level of vaginal incision to create vaginal opening of the uterovaginal canal was guided by an assistant from the vaginal end. A 2-cm transverse incision was then made at the uppermost portion of the vagina by the surgeon under guidance ( Fig. 3A ). Anterior and posterior vaginal flaps were thus created and identified. For the uterine stoma, a 2-cm sagittal incision was made on the lower end of the uterine corpus and extended caudally up to the atretic cervix and the endometrial cavity was exposed. Around 10 mL of hematometra was drained ( Fig. 3B ). A Hegar's dilator was introduced into the endometrial cavity through the uterine incision to delineate the existing canal. The endometrial canal was around 2 cm long in the sagittal plane. Posterior vaginal flap was modified (spatulated) to compensate for the disparity in the sizes of the muscular uterine stoma and relatively elastic and less muscular vaginal stoma. This was done by making a small longitudinal incision in the middle of the posterior vaginal flap perpendicular to its transverse edge.

**Fig. 4:**
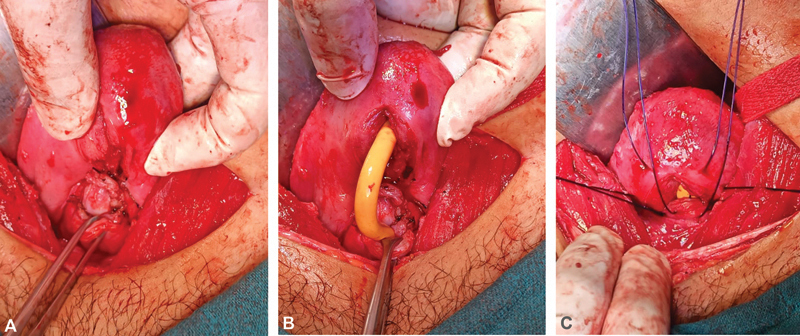
Construction of the uterovaginal canal. ( **A** ) Posterior wall of the canal formed by suturing posterior vaginal flap to the inferior margin of the hysterotomy incision. ( **B** ) A 20-Fr Foley's catheter inserted to provide support to the uterovaginal canal during and after its construction. ( **C** ) Circumferential apposition of rest of the vaginal flap and uterine edges to each other.

Construction of the neouterovaginal canal was started by first suturing the posterior flap of the vagina with the inferior margin of the uterine stoma using Polydioxanone monofilament suture (PDS II), which formed the posterior wall of the future uterovaginal canal with the fibrous raphe juxtaposed to it posteriorly ( Fig. 4A ). Horizontal mattress sutures were taken here to minimize tension along the suture line and to ensure continuity of the endometrium with the mucosal lining of the vagina. This continuity prevents fibrous tissue overgrowth during healing and stenosis of the anastomotic channel. 2 A 20-Fr Foley's catheter was then introduced into the uterine canal through the proximal vaginal stoma ( Fig. 4B ). With the Foley's catheter in situ, the rest of the uterine stomal edges were sutured circumferentially to the rest of the vaginal flap using horizontal mattress sutures ( Fig. 4C ). Slight anteversion of the uterus was observed after the procedure which is inevitable given the anatomy of the surgical reconstruction.

The postoperative period was uneventful. Foley's catheter was kept in situ for 4 weeks. She was given cyclical oral contraceptive pills for 3 months. A review hysteroscopy was done 3 months after the initial procedure to evaluate the outflow tract. The anastomotic channel was found patent and cylindrical, simulating a normal cervical canal. Healthy endometrial tissue was noted in the cavity in the proliferative phase. The patient reported to have been relieved of cyclical abdominal pain and having had cyclical menstrual periods for the last 6 months till the time this case is reported.

## Discussion and Conclusion

Understanding and delineating the exact anatomy of the Mullerian anomaly is the critical step before reconstruction or surgical correction of the genital tract is considered. An outflow obstruction at the level of cervix was evident in the present case on clinical examination. This was confirmed on MRI, which remains the most reliable investigation for diagnosis and surgical correlation of the defect morphology in Mullerian anomalies. 4 5 6

The ESHRE/ESGE classification for the present case was U0C4V0, which symbolizes a normal uterus (U0), an aplastic cervix (C4), and a normal vagina (V0). 7 This scheme, however, does not include detailed subclassification of the cervical anomalies. All cases of complete cervical aplasia together with defects of cervical formation such as cervical cord, cervical obstruction, fragmentation, etc. are collectively grouped under the subclass C4. The cervical dysgenesis classification described by Rock et al subcategorizes the development anomalies of cervix into three types. 2 Such categorization is important as clinical understanding of exact anatomical variation is crucial to form a definitive surgical plan and anticipate prognosis. The first type is constituted by a well-formed cervix with partial atresia of the endocervical canal at the level of cervical os. The second type is noted in the present case which is described to have a cervical remnant in the form of a fibrous cord of varying length and diameter with complete endocervical canal obliteration. In the third type, cervix is fragmented.

Management of cervicovaginal agenesis has evolved over the decades and extirpative procedures such as hysterectomy as a treatment option have fallen out of favor. In presence of sufficient cervical stroma, creation of a neocervical canal by drilling or coring with the insertion of a stent with or without grafting to expedite epithelization has been attempted successfully to relieve the obstructive menstrual symptoms. Chakravarty et al 8 described a novel reconstructive procedure in 18 patients with cervicovaginal atresia with hypoplastic cord such as cervix in which the lower uterine corpus was incised and the atretic cervix was vertically bisected anteriorly halfway through its thickness. Neocervix was constructed over a cervical stent by sequential rows of sutures taken over and across the cervical stent anteriorly, uniting the lower uterine corpus proximally and the ends of the bisected cervix distally. Canal remained patent during follow-up and menstruation was restored in all patients.

Canal reconstruction by digging through the native cervical remanent, however, carries overall higher rates of complications including restenosis, need for repeated surgeries, ascending infections, and even fatal peritonitis with septic shock. 3 When cervical reconstruction is not feasible, uterovaginal anastomosis is preferred and recently being recommended over other alternatives as the first treatment option, as it has a lower risk of restenosis, and successful reproductive outcomes have been reported with fewer complications. 9 10 The earliest series on uterovaginal anastomosis technique described by Deffarges et al 11 reported successful creation of outflow channel in 18 patients with failure due to restenosis in only 1 woman. They also reported six spontaneous conceptions in four of these women who delivered between 36 and 38 weeks' gestation via cesarean section. Subsequently, many successful open and laparoscopic approaches to uterovestibular anastomosis for cervicovaginal aplasia have been reported with good functional and reproductive outcomes. 9 10 12 13 14 15 16 17 Interestingly, mucinous secretions such as normal endocervical gland secretions have been noted during follow-up in patients, suggesting metaplasia of the most caudal endometrial glands in the neouterovaginal channel. 10 This possibly explains fewer rates of ascending upper genital tract infections reported in patients who undergo uterovaginal anastomosis.

**Fig. 5:**
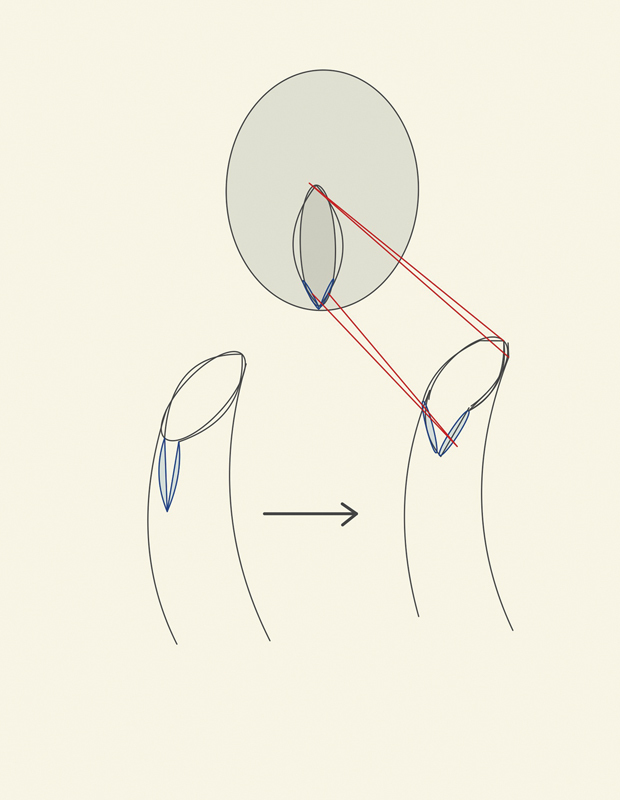
Spatulation of vaginal flap.

Spatulated anastomosis, instead of end-to-end attachment, was performed in the present case as the size and thickness of the uterine and vaginal stomas were different. The vaginal opening was fabricated in opposite obliquity to that of the hysterotomy incision and was further spatulated by giving a longitudinal incision in the middle of flap perpendicular to its transverse edge ( Fig. 5 ). Spatulation creates a wider area for anastomosis and is a standard technique in cardiovascular surgery for vascular anastomosis. 18 In the present case, spatulation improved the congruency between the two stomas during repair and enabled proper juxta position of uterine endometrium with vaginal mucosa. This is essential during construction of the anastomotic canal as discontinuity of the uterovaginal mucosa after repair may lead to fibrous tissue overgrowth leading to stenosis. 2

The main goals of surgery in our patient were to create an outflow tract and establish normal menstruation, which were satisfactorily achieved. The restoration of the tract patency renders a future possibility of using assisted reproductive technologies to achieve pregnancy. Sequelae of retrograde menstruation and endometriosis were not severe in our patient, highlighting importance of early diagnosis and timely treatment of the obstruction.

Early surgical correction with uterovaginal anastomosis should, therefore, represent the primary therapeutic option in patients with cervical agenesis or dysgenesis for most optimal outcomes.
